# The Microbiome of *Ehrlichia*-Infected and Uninfected Lone Star Ticks (*Amblyomma americanum*)

**DOI:** 10.1371/journal.pone.0146651

**Published:** 2016-01-11

**Authors:** R. T. Trout Fryxell, J. M. DeBruyn

**Affiliations:** 1 Department of Entomology and Plant Pathology, University of Tennessee, Knoxville, Tennessee, United States of America; 2 Department of Biosystems Engineering and Soil Science, University of Tennessee, Knoxville, Tennessee, United States of America; University of Kentucky College of Medicine, UNITED STATES

## Abstract

The Lone Star tick, *Amblyomma americanum*, transmits several bacterial pathogens including species of *Anaplasma* and *Ehrlichia*. *Amblyomma americanum* also hosts a number of non-pathogenic bacterial endosymbionts. Recent studies of other arthropod and insect vectors have documented that commensal microflora can influence transmission of vector-borne pathogens; however, little is known about tick microbiomes and their possible influence on tick-borne diseases. Our objective was to compare bacterial communities associated with *A*. *americanum*, comparing *Anaplasma/Ehrlichia* -infected and uninfected ticks. Field-collected questing specimens (n = 50) were used in the analyses, of which 17 were identified as *Anaplasma/Ehrlichia* infected based on PCR amplification and sequencing of *groEL* genes. Bacterial communities from each specimen were characterized using Illumina sequencing of 16S rRNA gene amplicon libraries. There was a broad range in diversity between samples, with inverse Simpson’s Diversity indices ranging from 1.28–89.5. There were no statistical differences in the overall microbial community structure between PCR diagnosed *Anaplasma/Ehrlichia-*positive and negative ticks, but there were differences based on collection method (*P* < 0.05), collection site (*P* < 0.05), and sex (*P* < 0.1) suggesting that environmental factors may structure *A*. *americanum* microbiomes. Interestingly, there was not always agreement between Illumina sequencing and PCR diagnostics: *Ehrlichia* was identified in 16S rRNA gene libraries from three PCR-negative specimens; conversely, *Ehrlichia* was not found in libraries of six PCR-positive ticks. Illumina sequencing also helped identify co-infections, for example, one specimen had both *Ehrlichia* and *Anaplasma*. Other taxa of interest in these specimens included *Coxiella*, *Borrelia*, and *Rickettsia*. Identification of bacterial community differences between specimens of a single tick species from a single geographical site indicates that intra-species differences in microbiomes were not due solely to pathogen presence/absence, but may be also driven by vector life history factors, including environment, life stage, population structure, and host choice.

## Introduction

In the southeastern United States, *Amblyomma americanum* (Lone Star tick) is the most frequently encountered tick species, likely responsible for a majority of tick bites [1,2]. It is the primary vector and amplifying reservoir of both *Anaplasma* and *Ehrlichia* species, which cause anaplasmosis and ehrlichiosis respectively [3]. Both are acute febrile diseases common in the U.S., and neither is transmitted transovarially (from female to offspring) in the tick host [4]. Anaplasmosis is commonly diagnosed in ruminants in the central and northeastern U.S. and is caused by infection with one or more *Anaplasma* species including *A*. *phagocytophilum*, *A*. *marginale*, and *A*. *odocoilei* [5]. Ehrlichiosis is primarily associated with canines [6] throughout the southeastern U.S. [7] and is caused by infection with one of several different bacteria including *E*. *chaffeensis*, *E*. *ewingii*, and Panola Mountain *Ehrlichia* [8–11]. Both are considered zoonotic diseases as they can be identified in wildlife, domesticated animals, and humans [2–14]. In Tennessee, *Anaplasma* and *Ehrlichia* species were identified in both questing and host-collected *A*. *americanum* [12–16].

While actively feeding, *A*. *americanum* ingest compounds from their host (blood, proteins) and simultaneously inject compounds into the host (anticoagulants, antihistamines, platelet aggregation inhibitors, histamine binding proteins, immune inhibitory proteins) [17,18]. This active feeding permits *A*. *americanum* to also harbor other pathogens. For example, the bacteria *Francisella tularensis*, which causes tularemia in humans [19], and the newly discovered Heartland virus, which thus far is pathogenic only in humans [20]. This tick also hosts non-pathogenic bacteria such as *Candidatus* Rickettsia amblyommii [21] and *Borrelia lonestari* [22]. Co-infections of multiple pathogens have been reported, but very few accurate rate estimates exist due to the design of standard diagnostic tests [15,23–26]. For example, the *groEL* gene is commonly amplified via nested PCR to positively identify both *Ehrlichia* and *Anaplasma* [27,28]; co-infections of the two are only identified when additional steps such as cloning and sequencing PCR products [29] or reverse line blot hybridization [30] are performed.

Ticks have a complex community of commensal organisms. *Amblyomma americanum* microbiome discovery studies have identified *Bradyrhizobium*, *Coxiella*, *Rickettsia*, and *Phenylobacterium* as dominant endosymbionts [31–34]. Changes in *A*. *americanum* bacterial community structure and diversity also occur following life events such as blood feeding and molting [33]. Research on other vectors has shown that vector microbiomes can have considerable influence on vector competence or the ability of a vector to transmit a pathogen [35,36]. Recently, a combination of microbiome culturing and sequencing studies identified bacteria that have important interactions with their vector-borne pathogens. For example: (1) Immune system development and parasite resistance of the tsetse fly were dependent on larvae harboring its endogenous microbiome during intrauterine development [36,37]. (2) *Plasmodium* development can be inhibited by bacteria in mosquito midguts, such that increased copies of gram-negative bacteria in *Anopheles* midguts was associated with lower *Plasmodium* infection rate and sporogonic-stage development [38–40]. Eliminating the microbiota within *An*. *gambiae* increased the ability of *P*. *falciparum* to colonize and replicate within the vector [39]. (3) Chikungunya virus influenced the diversity and composition of symbiotic bacteria in colony-raised *Ae*. *albopictus*: *Alphaproteobacteria*, *Bacteroidetes* and *Planctomycetes* abundances decreased with increased viral infection [41]. These studies provide promising evidence that symbiotic bacteria may modulate vector competence; however, very little research has focused on the tick microbiome and its relation to tick-borne diseases.

Pathogenic bacteria must compete with a diverse community of microflora (bacteria, fungi, protozoans and viruses) for resources and space in the tick vector [42,43]. The objective of this study was to test the hypothesis that within a single tick species (*A*. *americanum*) there would be differences in the bacterial communities associated with a pathogen (*Anaplasma*/*Ehrlichia* infected ticks) compared to uninfected ticks collected from the same geographical area (west Tennessee). Our goal was to identify potentially synergistic (present in positive ticks and absent in negative ticks) and antagonistic (absent in positive ticks and present in negative ticks) bacteria associated with *Anaplasma/Ehrlichia* infection and to compare their bacterial communities within field-collected questing *A*. *americanum*. We additionally evaluated the relationships between microbiomes and factors associated with collection (sex, trapping method, habitat type, and soil type).

## Materials and Methods

### Tick Collection

*Amblyomma americanum* specimens were collected from Ames Plantation (35.115366 N, -89.216735 W); a University of Tennessee managed research and education facility in western Tennessee. Previously reported specimens identified as *Ehrlichia*/*Anaplasma* positive or negative via PCR amplification of the *groEL* gene using *Ehrlichia/Anaplasma*-specific primers were used in this study [15]. Briefly, ticks were collected with a combination of vegetation drags and carbon dioxide baited traps from 13 different sites during 2012 (May–August). All ticks were stored in vials containing 80% ethanol, and identified to species, life stage, and sex using morphological keys [44–46]. The latitude and longitude for each collection was recorded which allowed for the soil type to be identified in ArcGIS 10.0 (ERSI, Redlands, CA) using data obtained from USDA Geospatial Gateway [47] and classified with county soil records [48,49].

### *Anaplasma* and *Ehrlichia* identification

All *A*. *americanum* adult specimens were screened for *Anaplasma* and *Ehrlichia* species by nested PCR amplification of *groEL* using primers specific to *Ehrlichia/Anaplasma* [15,27,28]; a method that amplifies both genera. Adult specimens were used in this study because they had already taken two blood meals giving them the greatest chance of acquiring one or more pathogens, along with potentially synergistic or antagonistic bacteria. Prior to DNA extraction, the tick was removed from ethanol and held overnight at room temperature in sterile water to allow any ethanol to diffuse from the tick into the water. Then, half of each specimen was subjected to total DNA extraction using the Fermentas Gene Jet Genomic DNA Purification Kit and protocol (Thermo-Fisher Scientific, Pittsburgh, PA), which yielded 140–720 ng of genomic DNA per specimen. Seventeen specimens had positive amplification [15]. Sanger sequencing of amplicons identified twelve of the seventeen as 97–100% identical to *E*. *ewingii* (GenBank KJ907744); two were 100% identical to Panola Mountain *Ehrlichia* (GenBank HQ658904); two were 99% identical to *A*. *odocoilei* (GenBank JX876642); and one was 99% homologous to *E*. *chaffeensis* (GenBank KJ907753). For clarity, the seventeen *Ehrlichia* and *Anaplasma* positive specimens identified by this standard PCR diagnostic method are referred to as ‘PCR-positive’ specimens.

### Specimen Selection

A total of 51 specimens were used in the analyses, of which 17 were PCR-positive and 34 were PCR-negative. An attempt was made to use twice as many negative specimens in the analyses to err on the side of identifying bacteria associated with pathogen infection while controlling for the different collection and environmental variables. There was no significant difference in number of PCR-positive specimens by pathogen, sex, trapping method, collection period, habitat type, or soil type (*P* > 0.05) (Table 1).

**Table 1:** The 51 *Amblyomma americanum* specimens used in the study did not differ by sex, trapping or collection method, collection month, habitat, or soil type (*P* > 0.05).

Collection Variable	PCR-negative	PCR-positive	Total
**Sex (*X***^***2***^ **= 1.42; df = 1; *P* = 0.234)**			
Male	8	6	24
Female	16	11	27
Total	34	17	51
**Trapping Method (*X***^***2***^ **= 0.0396; df = 1; *P* = 0.842)**			
CO_2_ trap	19	9	28
Drag	15	8	23
Total	34	17	51
**Collection Month (*X***^***2***^ **= 7.29; df = 3; *P* = 0.063)**			
(1) 10–16 May 2012	9	5	14
(2) 2–8 June 2012	9	0	9
(3) 27 June–6 July 2012	10	10	20
(4) 26 July–1 August 2012	6	2	8
Total	34	17	51
**Habitat (*X***^***2***^ **= 3.13; df = 3; *P* = 0.372)**			
Coniferous	13	4	17
Grasslands	7	3	10
Bottomland deciduous	0	1	1
Upland deciduous	14	9	23
Total	34	17	51
**Soil Type (*X***^***2***^ **= 1.53; df = 4; *P* = 0.822)**			
(1) Memphis silt loam	18	11	29
(3) Calloway silt loam	3	1	4
(4) Henry silt loam	8	4	12
(5) Gullied land complex	2	0	2
(7) Grenada-gullied land complex	3	1	4
**Total**	**34**	**17**	**51**

### Microbiome Analyses

The composition of the bacterial communities of each specimen was determined using Illumina sequencing of 16S rRNA gene amplicons. Extracted DNA was sent to the Hudson Alpha Bioinformatics Institute Genomic Services Laboratory (Huntsville, AL USA), where they amplified the V3-V4 region of the 16S rRNA gene with barcoded primers 341F and 785R [50]. Amplicon libraries were pooled and 250 base pair paired end sequence reads were obtained on the Illumina MiSeq platform. Reads were processed using the open source bioinformatic software package Mothur v 1.33.3 following the MiSeq SOP protocol [51]. Briefly, sequences with homopolymers longer than eight nucleotides or containing ambiguous bases were removed. Remaining sequences were aligned to a SILVA reference library and trimmed to 445 bases that started and ended at the same alignment position. The reads were subjected to the UCHIME chimera removal algorithm. Reads were classified using the Ribosomal Database Project database using at least 80% similarity to define taxonomy [52] and binned into operational taxonomic unites (OTUs) according to their taxonomic classification at the genus level (phylotype clustering). Sequences that classified as non-bacterial were removed. After screening, 3,428,296 reads remained, with a mean of 58,503 sequences per specimen. Sequences were deposited in MG-RAST (Project: TickAA_Ehrlichia; Accession No. 467501.3–467558.3).

### Data Analysis and Synthesis

Prior to diversity analysis of the tick microbiome communities, the number of sequences in each sample was normalized by randomly subsampling the number of sequences present in the smallest sample (13,508 reads) to eliminate the effect of uneven sampling depth on diversity estimation. Simpson’s Diversity index and richness were calculated using Mothur on this subsampled dataset. ANOVA was used to compare the mean diversity and richness by collection factor in R [53]. Libraries that contained operational taxonomic units (OTUs) classified as either *Ehrlichia* or *Anaplasma* were categorized as ‘MiSeq-positive’, those that did not have these OTUs were ‘MiSeq-negative’. This classification was important as the two methods (PCR amplification of *groEL* and Illumina high throughput sequencing) did not always agree. Thus, we categorized specimens using three different criteria: 1) specimens with *Ehrlichia/Anaplasma* based on the diagnostic PCR amplifying *groEL* (‘PCR-positive’); 2) specimens with *Ehrlichia/Anaplasma* in the Illumina libraries (‘MiSeq-positive’); and 3) specimen that were identified as *Ehrlichia/Anaplasma* positive using either approach (‘*Ehrlichia*-positive’).

To compare tick microbiome community structures, operational taxonomic unit (OTU) abundances were standardized by total OTUs in a sample to yield relative abundance. Relative abundances were square root transformed to down-weight high abundance OTUs. Spearman’s rank and Pearson correlation coefficients between individual OTU abundances and continuous variables were determined using R. To examine community structure, Bray-Curtis distances between samples were calculated and visualized with nonmetric multidimensional scaling using Plymouth Routines In Multivariate Ecological Research (PRIMER) v6 software (Lutton, UK) [54,55]. Analysis of similarity (ANOSIM) was used to determine if there was significant multivariate clustering of community structure based on collection factors. Taxa that were differentially represented according to collection factors or tick characteristics were identified using LEFSe [56], which reveals taxa that are significantly different in relative abundance between samples, and evaluates their contribution to explaining differences in community structure (effect size). Briefly, taxa that were differentially distributed between factors, as determined by a Kruskal-Wallis α > 0.05, were then used to build a linear discriminant analysis model; taxa that were discriminant between factors with logarithmic LDA scores > 2.0 were reported as differentially represented.

## Results

### Microbiome structure of *Amblyomma americanum*

**Fig 1:**
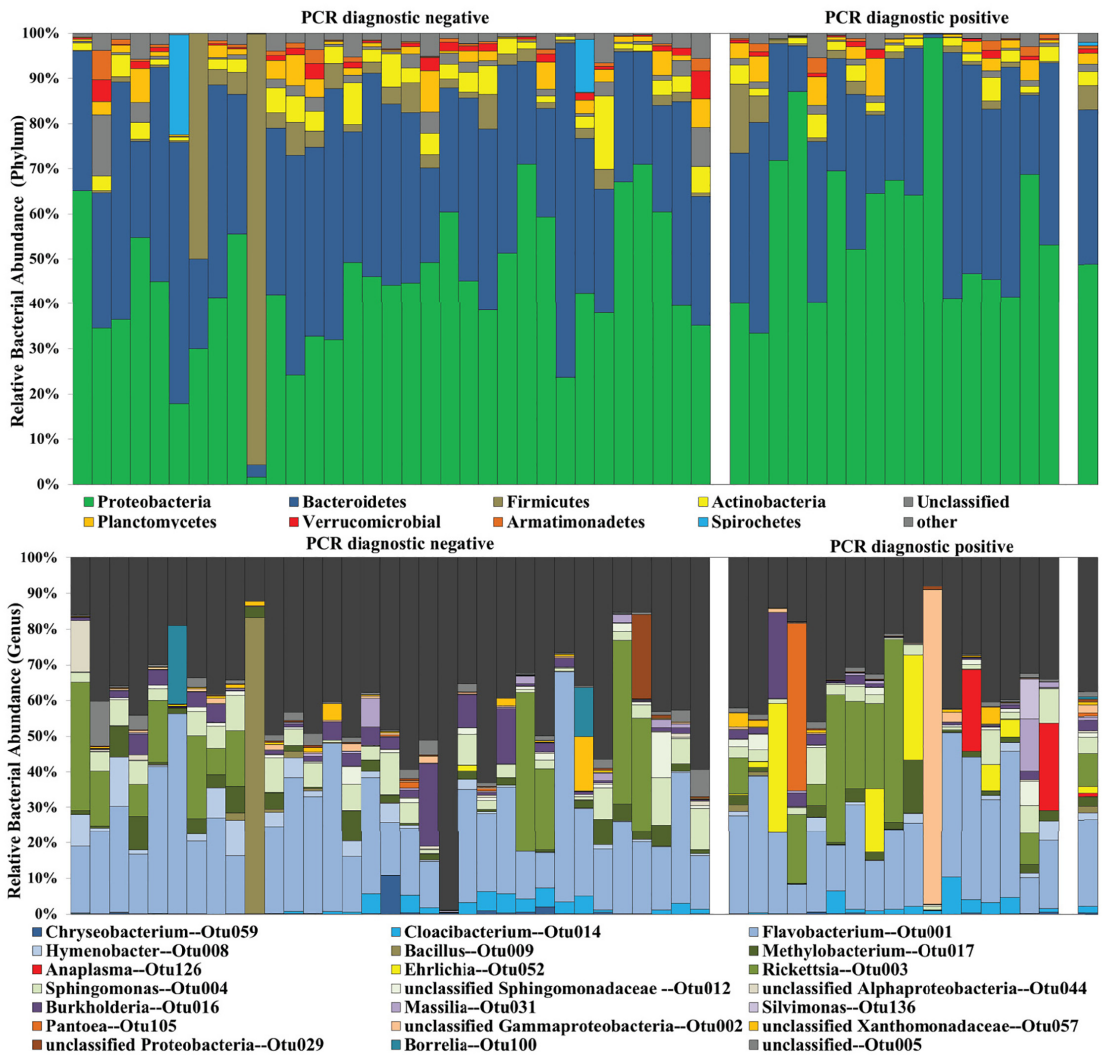
Bacterial biodiversity in PCR-positive and negative *Amblyomma americanum*. Relative abundance of taxa in the microbiome of 50 individual *Amblyomma americanum* collected in western Tennessee at the phylum (A) and genus (B) level. Specimens on the left were PCR-negative, those in the middle were PCR-positive. The final column is the mean relative abundances for all specimens (n = 50).

Of the 51 specimens submitted for analyses, 50 had libraries with sufficient coverage (99.7–99.9% coverage) for analysis. In total, 622 unique OTUs (classified to genus level) were identified across all libraries. Number of OTUs per library ranged from 80–288. There was a broad range in diversity between samples, with inverse Simpson’s Diversity indices ranging from 1.28–89.5 (Fig 1). OTUs belonging to phylum Proteobacteria (mean 49.3 ± 17.4% relative abundance) and Bacteroidetes (34.5 ± 14.0%) dominated the *A*. *americanum* microbiome (Fig 1). Other phyla in the microbiomes included Firmicutes (4.25 ± 13.4%), Actinobacteria (3.32 ± 2.68%), Planctomycetes (2.53 ± 2.44%), Acidobacteria (1.45 ± 1.26%), Verrucomicrobia (1.00 ± 1.2%). 1.6% of the reads were unclassifiable at 80% confidence to the RDP database. The remaining 2% were a variety of phyla comprising < 1% relative abundance. The most dominant OTUs were highly variable between specimens, but the most abundant (>1.00% relative abundance) across all specimens were *Flavobacterium* (24.4 ± 13.3%), an unclassified Gammaproteobacteria (2.22 ± 12.4%), *Rickettsia* (9.1 ± 14.5%), *Sphingomonas* (4.6 ± 3.6%), *Singulisphaera* (1.91 ± 1.81%), *Hymenobacter* (1.95 ± 3.00%), and *Bacillus* (1.86 ± 11.7%).

### Diagnostics

Of the 50 specimens, nested PCR of *groEL* genes using *Ehrlichia*-specific primers identified 17 specimens with *Ehrlichia* or *Anaplasma* (from here on these specimens are referred to as ‘PCR-positive’). Twelve of the 50 specimens contained OTUs classified as *Ehrlichia* or *Anaplasma* in their Illumina sequenced 16S rRNA gene libraries (from here on referred to as ‘MiSeq- positive’). Twenty specimens were identified as positive according to either PCR or 16S library sequencing (from here on referred to as ‘*Ehrlichia*-positive’). Six PCR-positive specimens were MiSeq-negative, while three PCR-negative specimens were MiSeq-positive. Therefore, combining the two approaches revealed a total of 20 specimens that were *Ehrlichia/Anaplasma*-positive. *groEL* PCR had a higher discovery rate (17 PCR positives / 20 total positives = 85%) than 16S rRNA gene libraries (14 OTU matches / 20 total positives = 70%); however, 16S rRNA gene sequencing additionally identified one specimen co-infected with both *Anaplasma* and *Ehrlichia*. This co-infection was not revealed by amplification and sequencing of *groEL*. The relative abundance of each OTU in that co-infected specimen was 0.36% for *Ehrlichia* and 0.01% for *Anaplasma*, highlighting the ability of this approach to identify bacteria at very low abundances.

### Microbiome differences between positive and negative ticks

**Fig 2:**
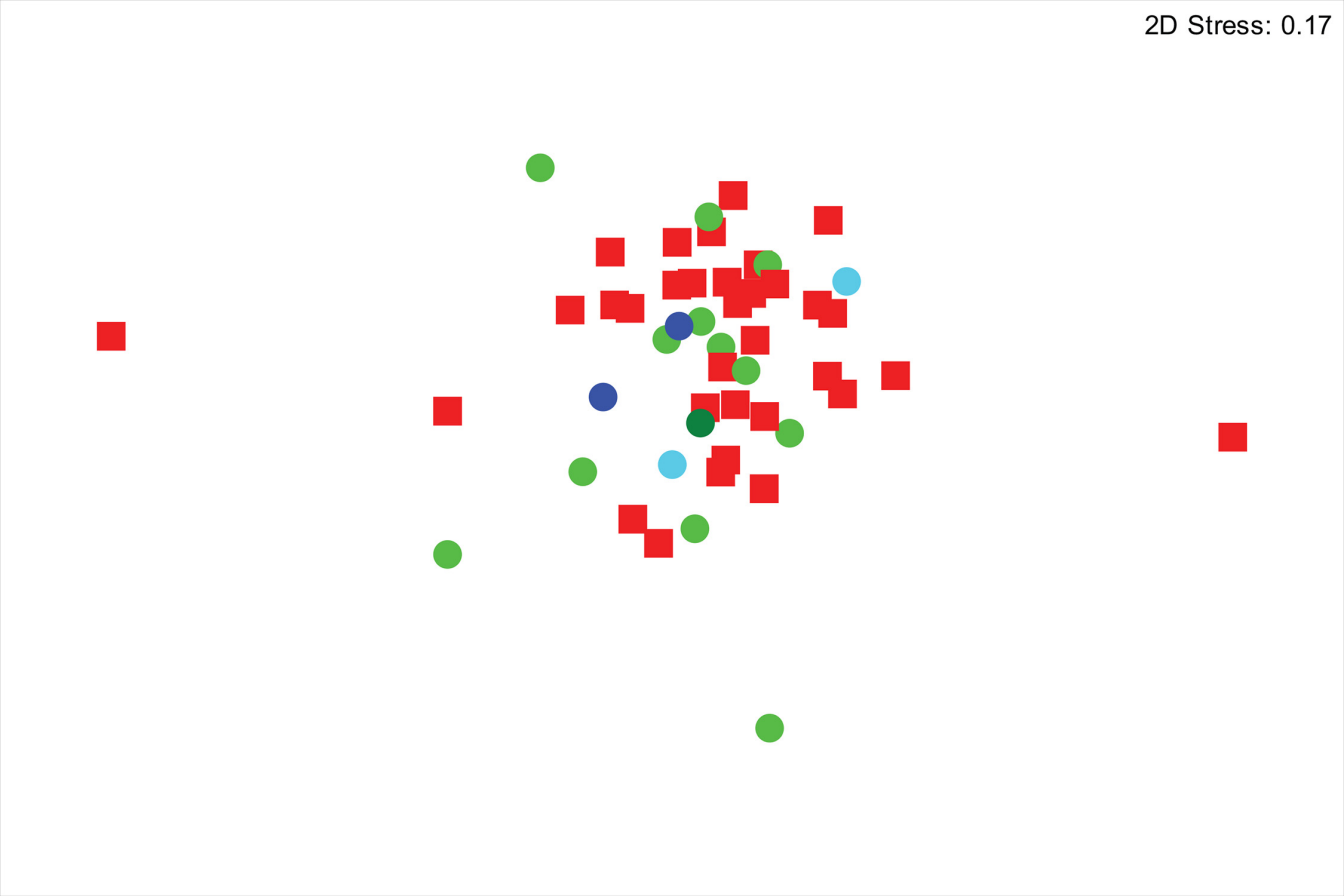
Bacterial community structure of PCR-positive and negative microbiomes. Nonmetric multidimensional scaling (NMDS) of Bray-Curtis distances shows no significant difference in microbiomes of PCR-positive (green and blue circles) and PCR-negative ticks (red squares). PCR positive ticks were previously identified with Sanger sequencing as having *Ehrlichia ewingii* (light green), *E*. *chaffeensis* (dark green), Panola Mountain *Ehrlichia* (dark blue), *Anaplasma odocoilei* (light blue).

**Fig 3:**
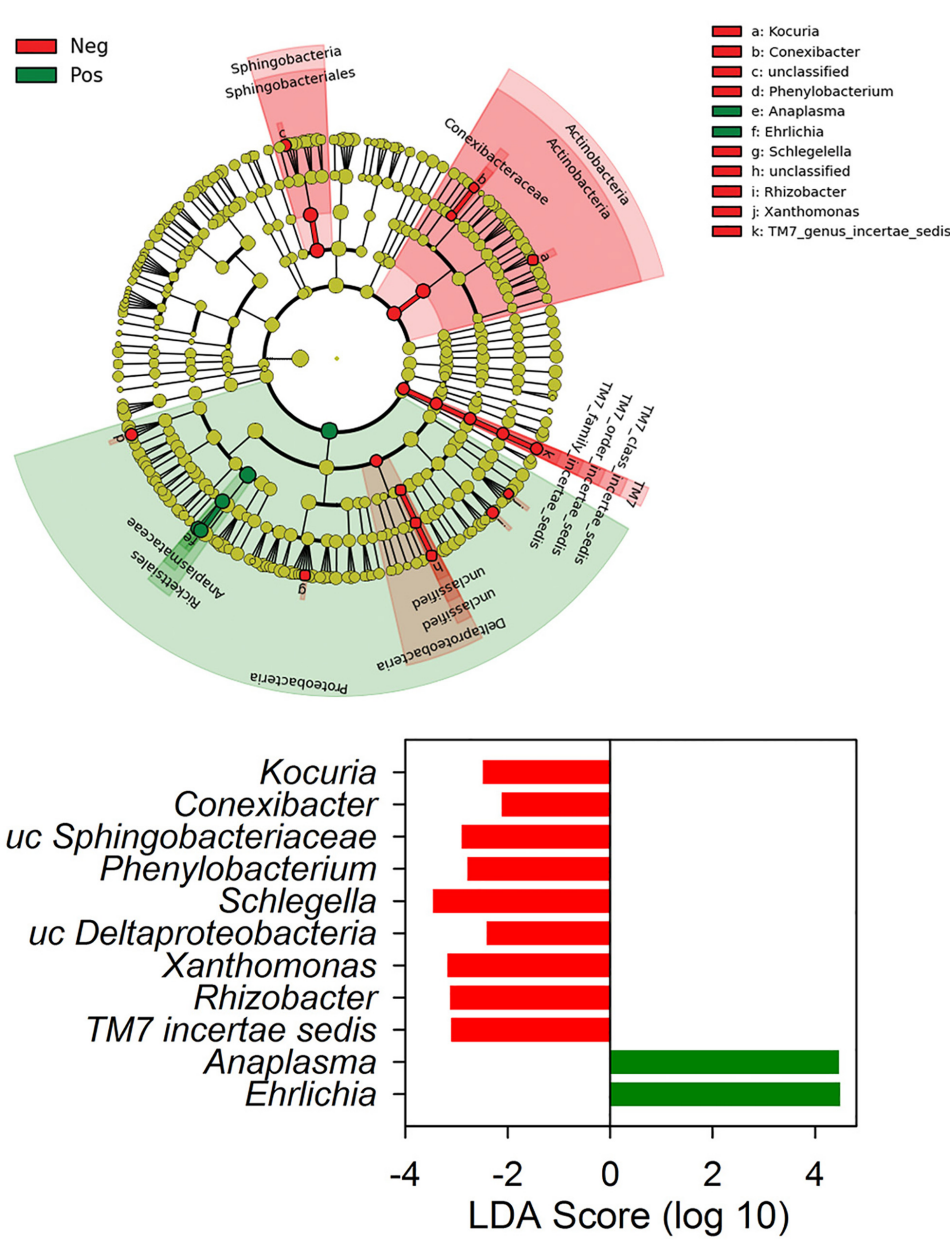
Significantly differentially represented bacterial taxa in Ehrlichia-infected ticks. Linear discriminant analysis (LDA) shows PCR-positive ticks had significant over-representation of *Ehrlichia* and *Anaplasma* (green) and PCR-negative ticks had an over-representation of a variety of taxa belonging to phyla Proteobacteria, Actinobacteria, Bacteroidetes and TM7 (red). Analysis was done using LEFSe, and phylogenetic relationship (A) and LDA scores (B) are shown.

There were no significant differences in richness, diversity, or community structure between PCR-negative and positive ticks (Fig 2, Table 2). In terms of phylum distribution, differences were not significant (T test *P* > 0.1). Despite no significant differences in phylum composition or community structure, there were significant differences between PCR positive and negative ticks in terms of differentially represented OTUs. A LEFSe discriminant analysis revealed that PCR positive and negative tick microbiome communities could be distinguished based on differences in the relative abundance of a few taxa. As expected, PCR positive ticks were characterized by significantly increased relative abundances of the Alphaproteobacteria *Ehrlichia* (OTU052) and *Anaplasma* (OTU126) in their microbiomes (Fig 3). OTU052 was detected in 9 of the 17 PCR-positive ticks with a mean relative abundance of 6.54 ± 11.8%. OTU126 was detected in 3 of the 17 PCR-positive ticks with a mean relative abundance of 2.79 ± 7.89%; in the two specimens that were identified via Sanger sequencing as infected with *Anaplasma odocoilei*, OTU126 made up 23.0% and 24.4% relative abundance. PCR-negative tick microbiomes had overrepresentation of several OTUs, belonging to phyla Proteobacteria, Actinobacteria, Bacteroidetes, and TM7 (Fig 3).

**Table 2:** ANOVA of tick microbiome richness (number of OTUs) and diversity (Simpson Index) indicated no significant differences by life history or collection factors ANOSIM comparison of community structure revealed significant differences (bolded) by sex, collection method, and soil type.

Factor	Levels	Richness #OTUs	Diversity (Simpson)	Structure ANOSIM
		F (*P*)	F (*P*)	Global R (*P*)
*Ehrlichia* (PCR diagnostic)	Positive, Negative	1.084 (0.303)	2.144 (0.150)	0.035 (0.264)
*Ehrlichia* (MiSeq OTUs)	Positive, Negative	0.126 (0.725)	1.067 (0.307)	-0.071 (0.808)
*Ehrlichia* (PCR or MiSeq)	Positive, Negative	0.519 (0.672)	0.802 (0.499)	-0.01 (0.523)
Sex	Male, Female	3.707 (0.06)	1.739 (0.194)	0.042 (0.054*)
Collection Method	CO_2_, Drag	0.109 (0.743)	0.745 (0.392)	0.07 (0.014**)
Habitat	Coniferous, Deciduous, Grassland	1.932 (0.156)	0.736 (0.484)	0.046 (0.146)
Soil Type	(1)Memphis silt loam (3)Calloway silt loam (4)Henry silt loam (5)Gullied land complex (7)Grenada-gullied land complex	3.279 (0.076)	0.228 (0.635)	0.291 (0.001***)

*P < 0.1

**P < 0.05

***P < 0.01

**Fig 4:**
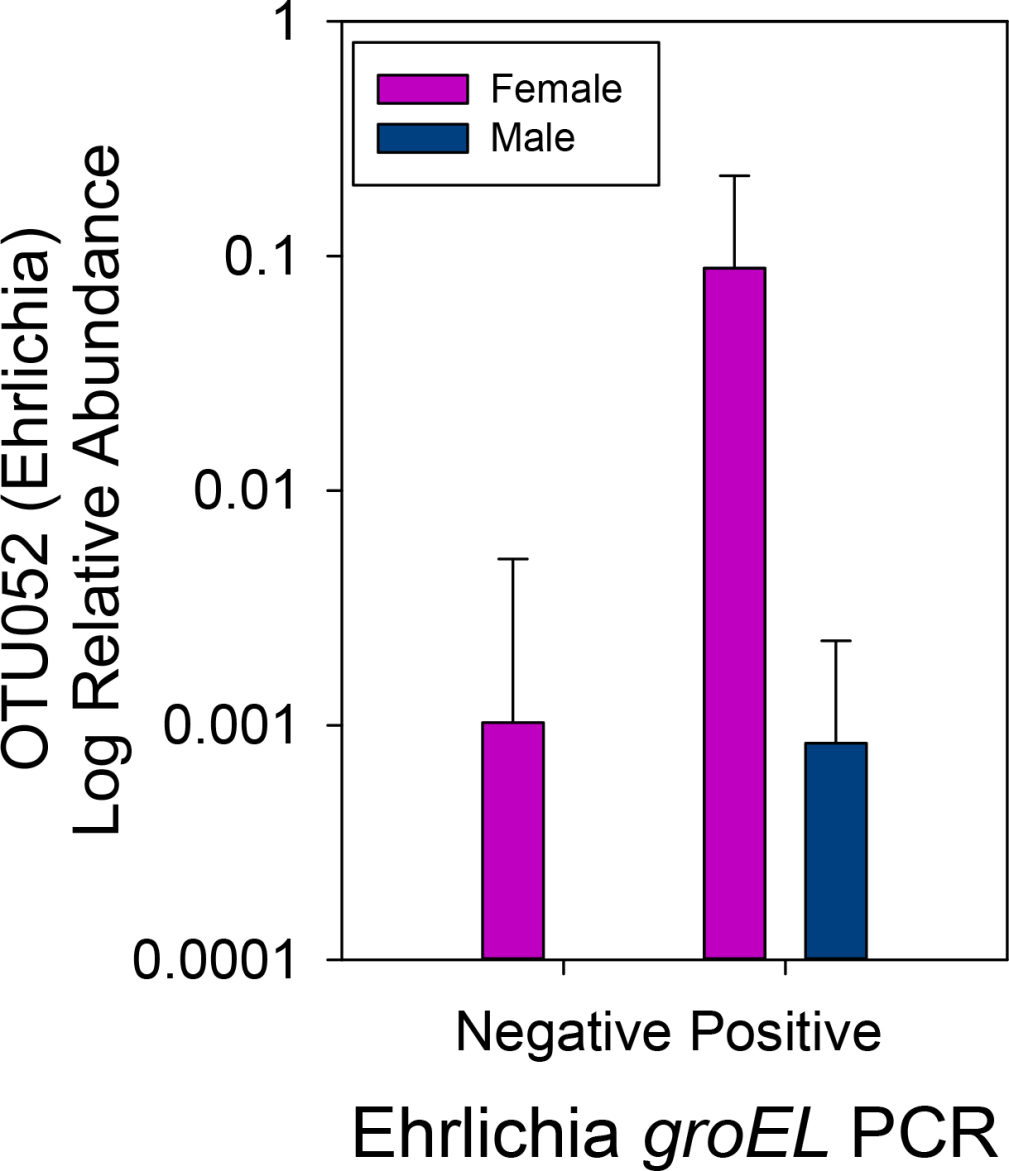
Female ticks had significantly higher relative abundance of *Ehrlichia* compared to males. The relative abundance of *Ehrlichia* (OTU052) in the 16S rRNA gene libraries was significantly higher in females compared to males (ANOVA *F* = 3.587, *P* = 0.064).

The relative abundance of OTU052 (*Ehrlichia*) was not correlated to community richness (r_s_ = 0.051, p = 0.723) or diversity (Simpson’s diversity index, r_s_ = 0.029, p = 0.842). It was also not correlated to the collection factors such as collection month (r_s_ = -0.114, p = 0.431). The only collection factor that was significantly associated with *Ehrlichia* abundance was sex; females carried a higher relative abundance of *Ehrlichia* than males (Fig 4). There was no significant difference in abundance between any of the other collection factors.

### Microbiome differences based on tick collection metadata

**Fig 5:**
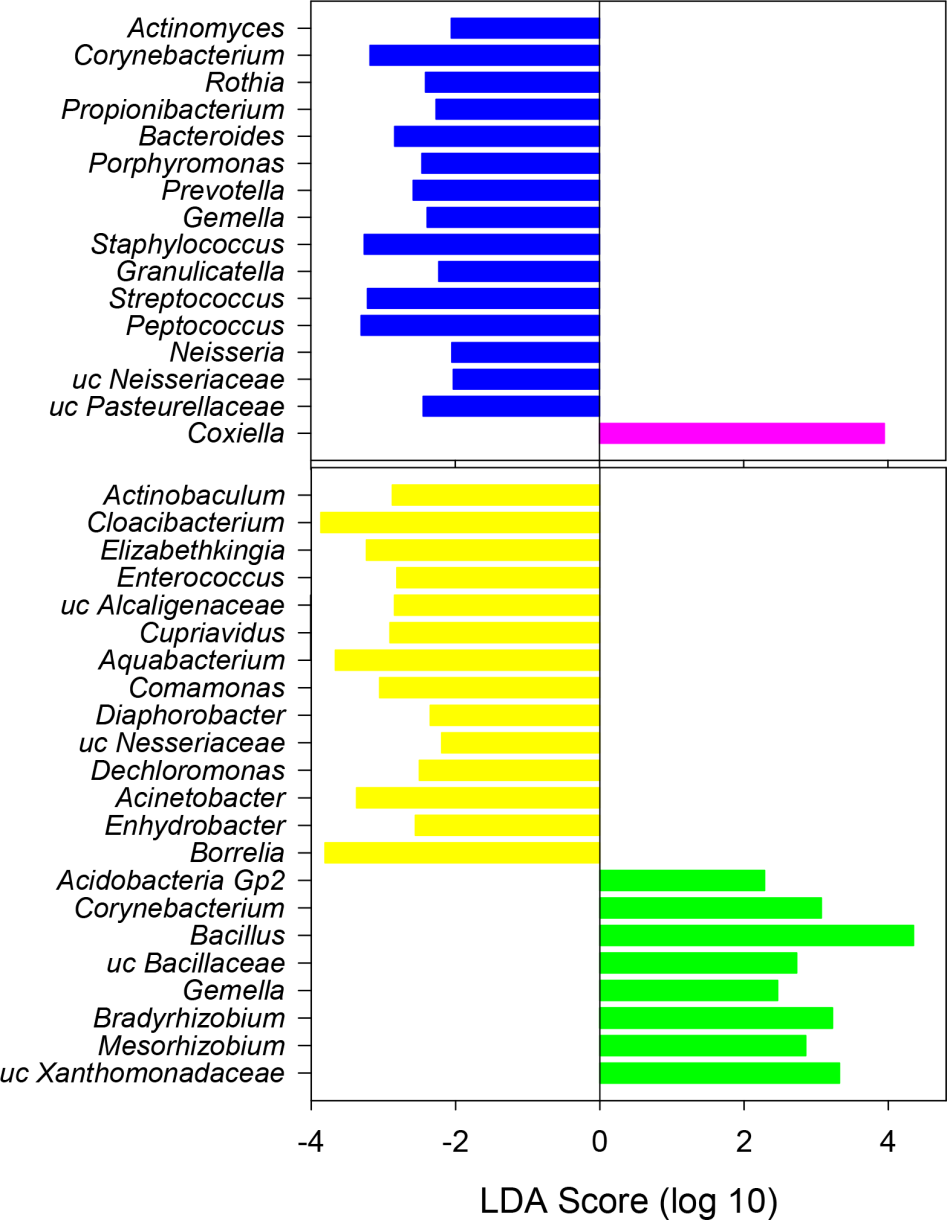
Bacteria genera associated with tick sex and collection methods. Taxa with significant effect sizes were identified for female and male ticks (A) and for collection methods (B) using linear discriminant analysis (LDA) in LEFSe. Females had significantly more *Coxiella* (pink) than males (blue), and CO_2_ trap collected ticks (yellow) had a different microbial composition than drag-collected ticks (green).

There was no significant difference in richness or diversity based on collection factors (Table 2); however, there was a significant difference in community structure based on several collection factors as determined by an ANOSIM analysis. Community structure was different between male and female ticks (Table 2); differentially represented taxa included *Ehrlichia* (mean relative abundance of 10.9% in females and 0.08% in males) (Fig 4) and *Coxiella* (1.99% in females and 0.09% in males). Interestingly, *Ehrlichia* and *Coxiella* were significantly and positively correlated across all specimens (r = 0.424, *P* = 0.002). Taxa overrepresented in male tick microbiomes were diverse, and included several Actinobacteria, Bacteroidetes, Firmicutes, and Proteobacteria (Fig 5A).

Tick microbiomes also differed slightly between those specimens collected questing to a CO_2_ trap and those specimens questing on vegetation to a drag (Table 2). Significantly and differentially represented taxa from specimens collected in traps included several Betaproteobacteria (*Burkholderiales*, *Neisseriales* and *Rhodocyclales*), Gammaproteobacteria, and a Spirochaete (*Borrelia*). Those specimens collected with a drag cloth had significantly greater abundances of Bacillales (Firmicutes) and Rhizobiales (Alphaproteobacteria) (Fig 5B).

We also noted a significant community structure based on soil type classification of collection sites (Table 2). Ticks collected from sites with soil type 4, characterized as a deep well drained to moderately well drained, medium texture soils in bottomland deciduous habitat (e.g. Henry silt loam), had a significantly different microbiome structure compared to those from upland areas with well-drained soil types (e.g. silty soil on upland flats, sandy soils, Calloway silt loam, Guillied land complex, Memphis silt loam) [15,48–49]. According to a LEFSe LDA no single taxa had a large enough effect size to explain the differences between soil type 4 and the other soils types; instead the differences are driven by a high variability in the tick microbial community structures from soil type 4 compared to the other locations (data not shown).

### Genera of potential pathogenic importance

**Fig 6:**
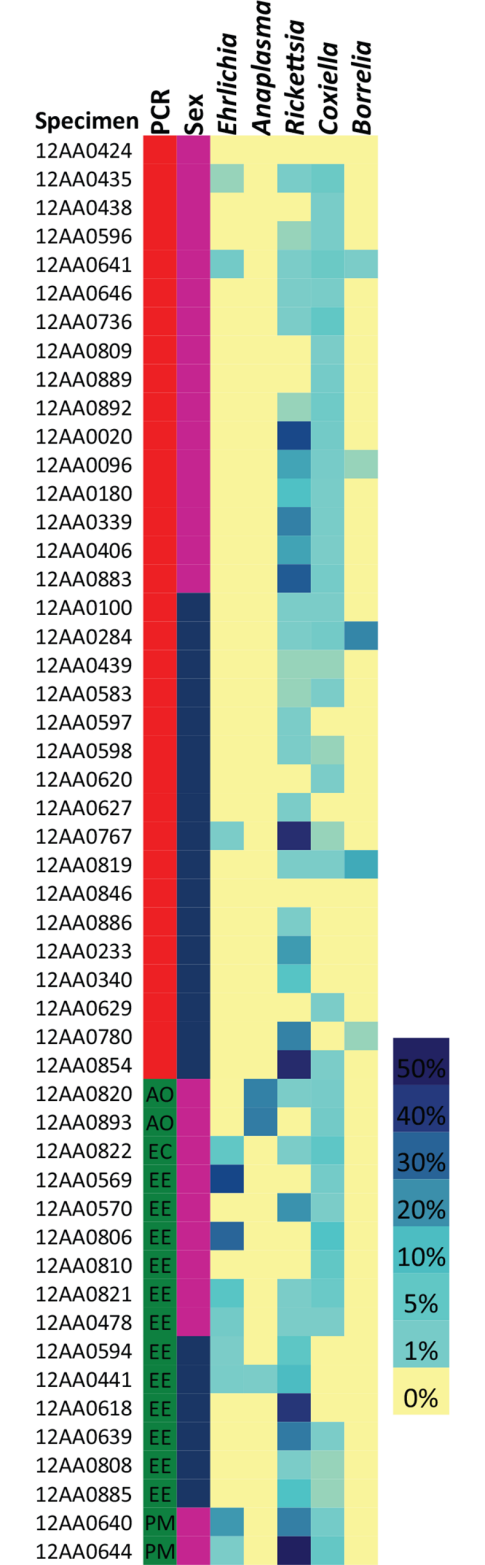
*Anaplasma*, *Borrelia*, *Coxiella*, *Ehrlichia*, and *Rickettsia* were identified in *Amblyomma americanum*. Heat map shows relative abundance of OTUs of public health importance. It also reveals co-infections in 15 of the specimens, as well as additional *Ehrlichia* and *Anaplasma* infections missed by PCR amplification of *groEL* genes. Specimen = tick identification number; PCR = red are negative and green are positive via PCR amplification of *groEL* with *Ehrlichia*-specific primers. Sanger sequencing of groEL amplicons provided taxonomic information: AO = *Anaplasma odoicoili*, EC = *Ehrlichia chaffeensis*, EE = *E*. *ewingii*, PM = Panola Mountain *Ehrlichia*). Sex = pink are female and blue are males.

Other bacterial genera of potential public health importance were identified in the 16S rRNA gene libraries (Fig 6). Known tick-associated bacteria were identified in many specimens, including *Borrelia* (n = 5), *Coxiella* (n = 39), and *Rickettsia* (n = 39) (Table 3). Other bacteria of potential interest identified in the libraries included *Bacillus*, *Burkholderia*, *Legionella*, *Pseudomonas*, *Schlegelella*, *Staphylococcus*, and *Streptococcus*. *Pseudomonas* and *Streptococcus* were identified in all 50 samples at comparatively high relative abundances (> 5.9%); *Bacillus*, *Burkholderia*, and *Staphylococcus* were identified in more than 80% of the specimens.

**Table 3:** Illumnia sequencing of the 16S rRNA gene V3-V4 region amplified two potential pathogenic tick bacteria and three known tick-associated bacteria. Means and standard errors (SEM) are presented.

OTU #	Genus	Relative abundance (all specimens)		Prevalence (% of specimens)	Mean Relative Abundance in MiSeq-Positive Ticks (±SEM)
		Range	Mean (±SEM)		
Potential Bacterial Pathogens					
126	*Anaplasma*	0.01%–24.4%	0.9% (±0.66%)	3 (6%)	15.8% (±7.91%)
052	*Ehrlichia*	0.01%–36.3%	2.0% (± 0.99%)	12 (24%)	8.3% (±3.65%)
Tick-Associated Bacteria					
100	*Borrelia*	0.01%–22.0%	0.7% (± 0.52%)	5 (10%)	7.2% (±4.58%)
090	*Coxiella*	0.01%–8.6%	1.1% (± 0.27%)	39 (78%)	1.4% (±0.32%)
003	*Rickettsia*	0.01%–51.2%	9.1% (± 2.05%)	39 (78%)	11.7% (±2.49%)

### Co-infection

Co-infections were identified in 15 of the ticks (Fig 6). 16S rRNA gene libraries revealed ten specimens infected with both *Ehrlichia* and *Rickettsia*. All five specimens that contained *Borrelia* also had *Rickettsia*, but none of those five specimens were infected with *Ehrlichia* or *Anaplasma*. Another specimen was infected with a *Rickettsia* and *Anaplasma*. Two ticks contained three genera of interest; one had *Rickettsia*, *Ehrlichia*, and *Borrelia*; and a second had *Rickettsia*, *Ehrlichia*, and *Anaplasma*.

## Discussion

The most commonly identified bacteria within *A*. *americanum* belonged to the phyla Proteobacteria (e.g. *Rickettsia*, *Sphingomonas*), Bacteroidetes (e.g. *Flavobacteria* and *Hymenobacter*), and Firmicutes (e.g. *Bacillus*). Contrary to our hypothesis, there was no significant difference in the overall microbiome bacterial community structure between negative and positive ticks; however, positive ticks were characterized by increased relative abundances of *Ehrlichia* and *Anaplasma* in their microbiomes, corroborating the results from the nested PCR assay. Several bacteria were identified with significantly higher relative abundance in PCR-negative ticks, including *Rhizobacter*, *Xanthomonas*, *Schlegelella*, *Phenylobacterium*, *Conexibacter* and *Kocuria* (Fig 3). It was also noted that *Borrelia* was only present in PCR-negative specimens (n = 5). These bacterial taxa may be potentially antagonistic with *Ehrlichia* or *Anaplasma;* however, experimental validation is needed to reveal microbial interactions between these organisms.

*Coxiella* and *Ehrlichia* were identified in significantly higher relative abundances in female ticks compared to males. *Coxiella* has been found in all tick tissues with large abundances in tick ovaries [57]; elevated *Coxiella* abundances in females has also been identified in the tick *Rhipicephalus microplus* [58]. In addition, the relative abundance of *Ehrlichia* was significantly and positively correlated to *Coxiella* across all specimens. We do not know the mechanism behind this association. Both *Coxiella* and *Ehrlichia* are Gammaproteobacteria, which are often considered medically and ecologically important bacteria. *Coxiella* has been previously identified in *A*. *americanum* and speculated to be an obligate endosymbiont because it was found at 100% frequency in a number of *A*. *americanum* studies from different locations [32], has a reduced genome [32], is vertically transmitted [18], and was amplified from all *A*. *americanum* life stages [32,33]. Nonpathogenic members of vector microbiomes are of considerable interest in terms of modulating pathogens, through competition, gene transfer, or other mechanisms. For example, *A*. *americanum* can harbor *Coxiella* spp. endosymbionts, which are closely related to the highly pathogenic *C*. *burnetii*, the causative agent for Q fever. It has been demonstrated that *C*. *burnetii* originated from a nonpathogenic *Coxiella* endosymbiont via horizontal gene transfer and convergence [59]. In our study, it is possible that the *Coxiella* identified in the libraries is an obligate endosymbiont, but it is interesting to note that it was only identified in 74% of our *A*. *americanum* specimens. This suggests that even if *Coxiella* is dependent on the tick host, the tick host may not be dependent on *Coxiella* (i.e. it is not an obligate endosymbiont). The history of both convergence and horizontal gene transfer in the evolution of *C*. *burnetii* [59] indicates the importance of discovering the microbial community within vectors.

In this study, the goal was to examine microbial differences between infected and uninfected ticks. In order to ensure any identified differences were due to associations with or without the pathogenic bacteria and to minimize potentially inherent differences, specimens were selected that were as similar as possible in terms of collection metadata (sex, habitat) and sampling scheme (site, collection method). To our surprise, we observed that the microbiome community structure varied depending on how and where each specimen was collected. We were also surprised to find that habitat (*P* = 0.146) did not matter nearly as much as soil type (possibly due to contamination, *P* = 0.001) and collection method (*P* = 0.014). To our knowledge, this is one of the first studies to demonstrate microbial differences based on collection method. This is important as it indicates that specimen selection and documentation of ecological metadata is critical for future microbial studies. It is known that ticks spend a majority of their life in leaf litter off their host, providing opportunity for the environment to structure the microbiome, either indirectly (via changes in abiotic parameters) or directly (via incorporation of microbes). In our study, ticks were stored in ethanol and then given a water bath, but the exoskeleton or outer surface of the tick was not additionally sterilized prior to DNA extraction, so the relationship to soil type may be partially due to soil contamination on the outside of the tick. We also revealed different microbiome structures between ticks collected by CO_2_ trap and those collected by dragging. This also supports the idea that environment is structuring the microbiome: a tick questing towards a trap in the vegetation (i.e. to a resting host) is exposed to a different environment than a tick questing on or above the vegetation (i.e. to an active host). An alternative explanation for the differences between trapping methods is that there are members of the microbiome that influence questing behavior [60,61]. Previous studies have shown that the microbiome of arthropod vectors was more influenced by by the type of vector (fleas vs ticks) than by host or environment [62], and that bacterial communities are highly structured by host species [63]. It has also been shown that blood feeding and molting result in significant microbiome changes in *A*. *americanum* [33]. Here, we provide some early evidence that the environment may structure vector microbiomes. Additional research should focus on identifying the time points in the tick life cycle when microbial communities are established, with a particular focus on those stages where exogenous microbes are most likely to be introduced (i.e. molting and feeding stages). These different tick life events likely influence the tick’s microbiome, and therefore may play a role in modulating pathogen establishment and/or transmission.

An unexpected outcome of this study was that the two approaches used (traditional PCR-based diagnostics and Illumina 16S rRNA gene library sequencing) did not always yield the same taxa identification. Sensitivity comparisons between the two assays indicated that nested PCR of *groEL* was slightly better at identifying *Ehrlichia/Anaplasma* in these specimens. However, 16S library sequencing was able to identify co-infections and additional bacteria of interest that cannot be identified with traditional gene PCR and targeted sequencing. Therefore, 16S sequencing in conjunction with traditional PCR assays could improve diagnostic results. The major limitation of Illumnia 16S sequencing was the short reads (ca. 400bp), which make it difficult to classify at the species or strain level. For example, Sanger sequencing of *groEL* genes differentiated several *Ehrlichia* and an *Anaplasma* species (4 genotypes), whereas Illumnia sequencing only differentiated specimens in terms of *Ehrlichia* or *Anaplasma* genera (2 genotypes). These diagnostic discrepancies add to our questions regarding pathogen transmission. Combining Illumina 16S sequencing with quantitative PCR may help determine minimum infection rates of an infected tick. Multiple approaches are needed to ultimately reveal the ecological interactions between pathogenic bacteria and the other members of the tick microbiome.

While vector microbiome work is still beginning, the questions are continuously evolving. In our attempts to identify antagonistic or synergistic bacteria associated with the presence or absence of a pathogen we unveiled new findings of the importance of sampling and specimen selection. The specimens examined in this study were similar to other studies in regards to microbiome composition, frequency, and coinfections [34]; however, our study design additionally allowed us to delve deeper into influences of life history and environment. These unexpected discoveries add to our outstanding questions regarding tick microbial ecology and the role the microbiome has in tick life histories and pathogen transmission. Can we, or will we, define specific tick endosymbionts that correlate directly or inversely with infection and transmission of specific pathogens, e.g. [64]? Will a bacteria be identified that can be used for future tick or tick pathogen management options such as employing paratransgeneis for tick control, e.g. [65]? Additionally, these methods will also be useful for the discoveries surrounding the other members of the tick microbiome, e.g. viruses [66]. The identification of bacterial community differences between specimens of a single tick species from a single geographical site leads us to hypothesize that the intra-species difference in microbiome structure may not be due solely to pathogen presence/absence, but are also likely driven by tick life history factors, including environment, life stage, population structure, and host choice.
