# Correction: The Microbiome of *Ehrlichia*-Infected and Uninfected Lone Star Ticks (*Amblyomma americanum*)

**DOI:** 10.1371/journal.pone.0155559

**Published:** 2016-05-09

**Authors:** R. T. Trout Fryxell, J. M. DeBruyn

The Data Availability Statement for this paper is incorrect. The correct statement is: Data are available at MG-RAST (4676501.3–4676559.3), project 16202.
